# Supplementary material for: Increase in Cape Verde hurricanes during Atlantic Niño
Source: Nat Commun. 2023 Jun 22;14:3704. doi: 10.1038/s41467-023-39467-5 (PMC10287659; doi:10.1038/s41467-023-39467-5)
Supplement: Supplementary file 1 — Supplementary information [file 41467_2023_39467_MOESM1_ESM.pdf]

Supplemental Information

Increase in Cape Verde hurricanes during Atlantic Niño

Dongmin Kim[1,2], Sang-Ki Lee[2], Hosmay Lopez[2], Gregory R. Foltz[2], Caihong Wen[3], Robert West[4,2] and Jason Dunion[1,2]

[1]Cooperative Institute for Marine and Atmospheric Studies, University of Miami, Miami, FL, USA

[2]Atlantic Oceanographic and Meteorological Laboratory, NOAA, Miami, FL, USA

[3]Climate Prediction Center, NOAA, College Park, MD, USA

[4]Northern Gulf Institute, Mississippi State University, MS, USA

Included materials:

Supplementary Tables 1

Supplementary Figures 1, 2, 3, 4, 5, and 6

Table S1. **Atlantic Niño/Niña, El Niño/La Niña and positive/negative Atlantic meridional mode years used in this study.** Atlantic Niño events are identified based on the criterion that the area-averaged sea surface temperature anomalies (SSTA) over the Atlantic Niño 3 region (ATL3, 3S° - 3°N, 20°W - 0°) exceeds one standard deviation (σ = 0.32 K) during June-November. Atlantic Niña events are defined using the criterion that ATL3 SSTA is less than -σ. The same criteria are applied for El Niño-Southern Oscillation based on the area-averaged SSTA over the NINO3.4 region (σ = 0.50 K; 5S° - 5°N, 170°E - 120W°). Similarly, (+)/(-) Atlantic meridional mode (AMM) years are identified when the normalized AMM index during June-November exceeds one standard deviation (positive AMM) or is less than negative one standard deviation (negative AMM).

Atlantic Niño (n=19)	Atlantic Niña (n=14)	El Niño (n=17)	La Niña (n=21)	(+) AMM (n=15)	(-) AMM (n=18)
1949, 1951, 1960, 1963, 1966, 1968, 1973, 1981, 1984, 1987, 1988, 1995, 1998, 1999, 2003, 2008, 2010, 2016, 2021	1958, 1964, 1965, 1967, 1969, 1971, 1976, 1978, 1982, 1983, 1992, 1994, 2005, 2013	1951, 1953, 1957, 1963, 1965, 1969, 1972, 1976, 1977, 1982, 1987, 1991, 1994, 1997, 2002, 2009, 2015	1950, 1954, 1955, 1956, 1964, 1970, 1971, 1973, 1975, 1984, 1985, 1988, 1998, 1999, 2000, 2007, 2010, 2011, 2016, 2020, 2021	1952, 1955, 1958, 1980, 1995, 1998, 2001, 2004, 2005, 2006, 2010, 2011, 2012, 2013, 2017	1963, 1965, 1968, 1971, 1972, 1973, 1974, 1975, 1977, 1982, 1984, 1986, 1991, 1993, 1994, 2000, 2002, 2018

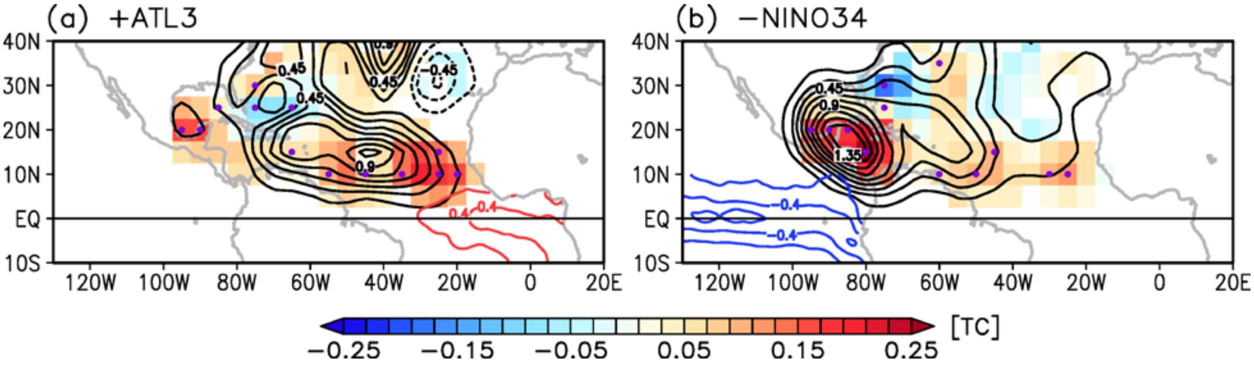

Fig. S1. Impact of Atlantic Niño and El Niño-Southern Oscillation on Atlantic tropical cyclone activity and associated large-scale environment during the post-satellite period (1979-2021). Partial regressions of tropical cyclone genesis (shaded), track density (black contours), and sea surface temperature anomalies (red and blue contours, interval is 0.2 K) onto (a) Atlantic Niño (ATL3) and (b) Niño 3.4 (NINO34) indices for the post-satellite period (1979-2021).

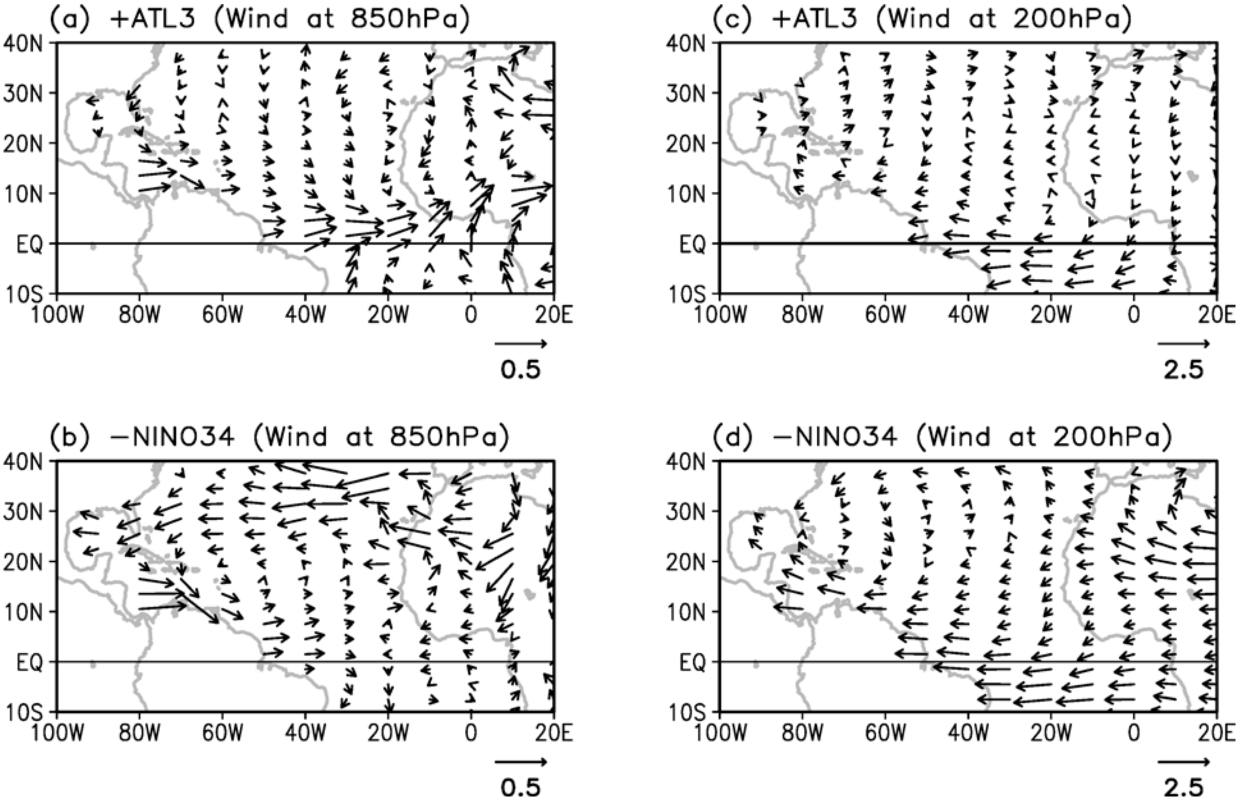

Fig. S2. Partial regression analysis of upper- and low-level wind anomalies on Atlantic Nino/Nina and El Niño-Southern Oscillation. Partial regressions of wind at 850 hPa onto (a) Atlantic Niño (ATL3) and (b) Niño3.4 (NINO34) indices for 74 years (1948-2021). (c) and (d) are the same as (a) and (b) but for wind at 200 hPa. The sign of NINO3.4 is reversed, and the regressed wind vectors over the Americas and Pacific Ocean are masked out.

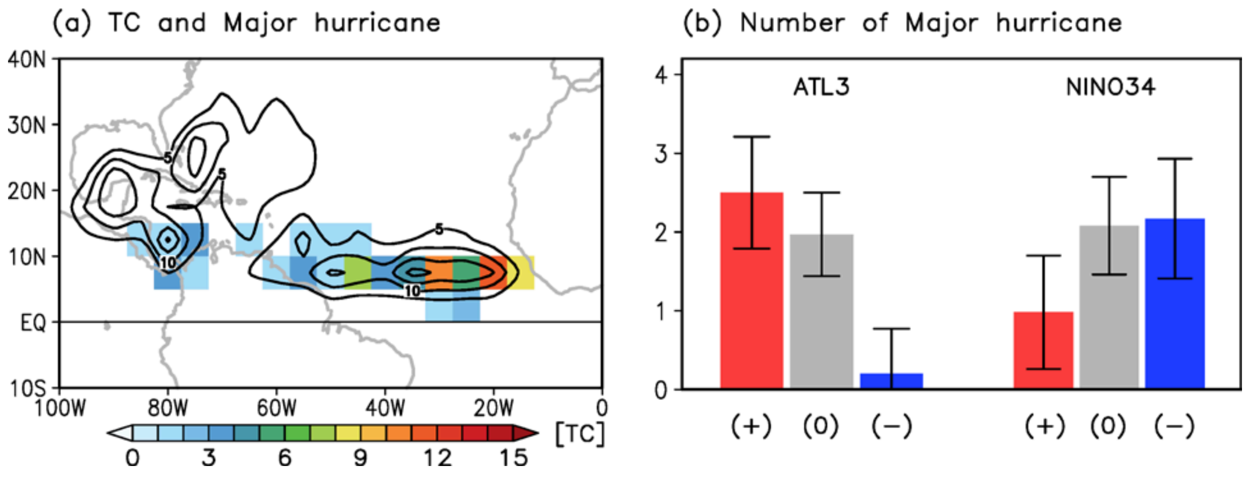

Fig. S3. Impact of Atlantic Niño/Niña and El Niño-Southern Oscillation on the genesis of major hurricanes in the tropical North Atlantic during the post-satellite period (1979-2021). (a) Spatial patterns of tropical cyclone (TC) genesis for all-category (contours, total number of TC genesis , intervals of 5 TC per contour line) and category 3-5 TCs (shaded, total number of category 3-5 hurricane genesis) for the post-satellite period (1979-2021). (b) The number of major hurricanes (category 3-5) formed over the tropical North Atlantic (60°W-10°W, 5°N-20°N) during positive (red bars), neutral (grey bars), and negative (blue bars) phases of (left) Atlantic Niño (ATL3) and (right) Niño 3.4 (NINO34) indices for the post-satellite period (1979-2021). The error bars indicate the 95% confidence level based on a two-tailed Student's t-test.

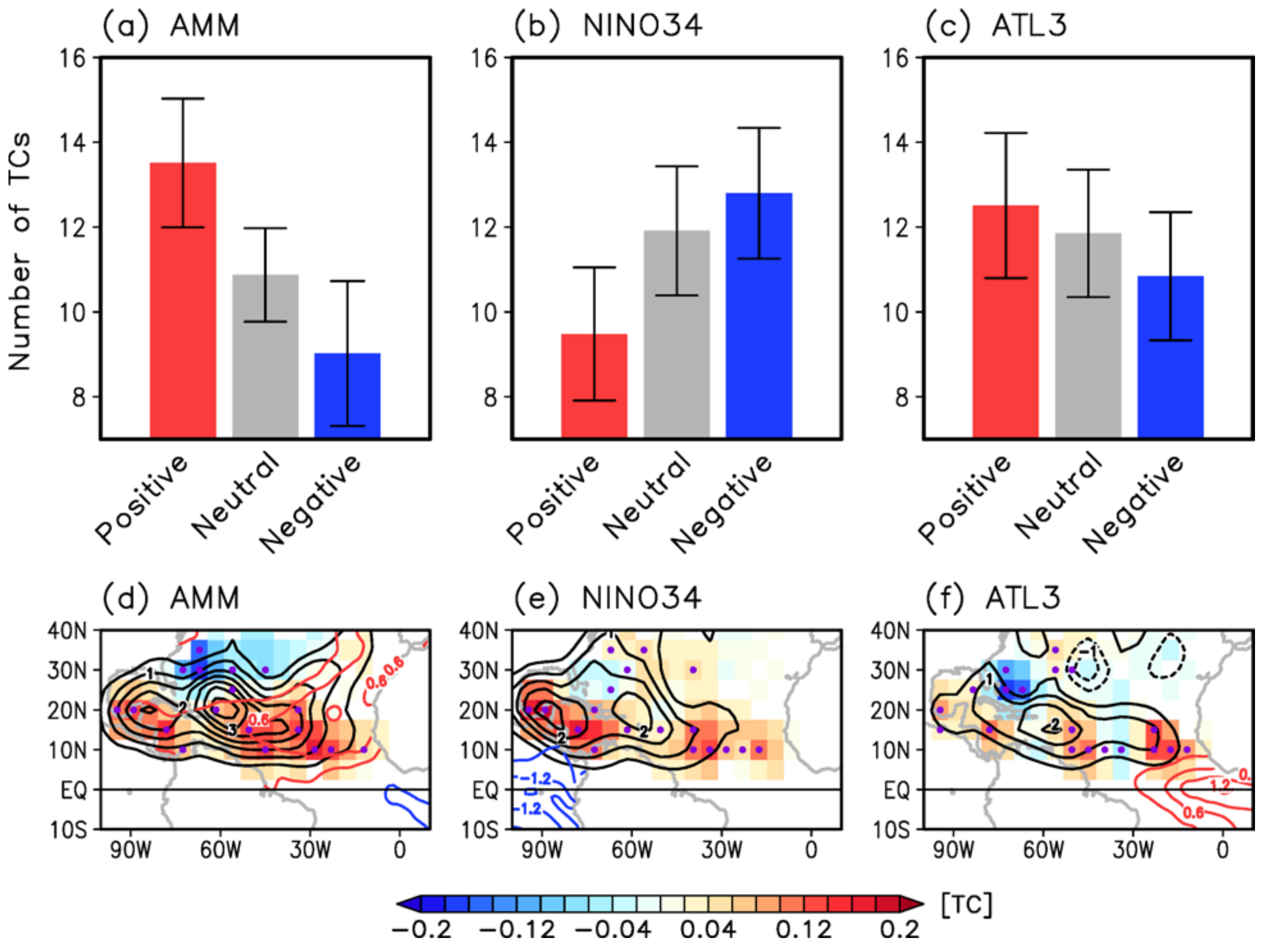

Fig. S4. Composite analysis of Atlantic tropical cyclone activity associated with Atlantic meridional mode, El Niño-Southern Oscillation, and Atlantic Niño/Niña. (a) Number of June-November tropical cyclones (TC) over the Atlantic during positive Atlantic meridional mode (AMM, red bar), neutral (gray bar), and negative AMM (blue bar) years for 74 years (1948-2021). (b) is the same as (a) but for El Niño (red bar), neutral (gray bar), and La Niña (blue bar). (c) is the same as (a) but for Atlantic Niño (red bar), neutral (gray bar), and Atlantic Niña (blue bar), respectively. Error bars indicate the 95% confidence level based on a two-tailed Student's t-test. (d) Difference in TC genesis (shaded, per year), track density (black contours, intervals of 0.5 TCs per year), and sea surface temperature anomalies (red and blue contours,

intervals of 0.3 K) between positive and negative AMM. (e) is the same as (d) but between La Niña and El Niño. (f) is the same as (d) but between Atlantic Niño and Atlantic Niña. Purple dots indicate where the difference in TC genesis is significant above the 95% confidence level based on a two-tailed Student’s t-test. TC genesis and track density are spatially smoothed to aid visual comparison.

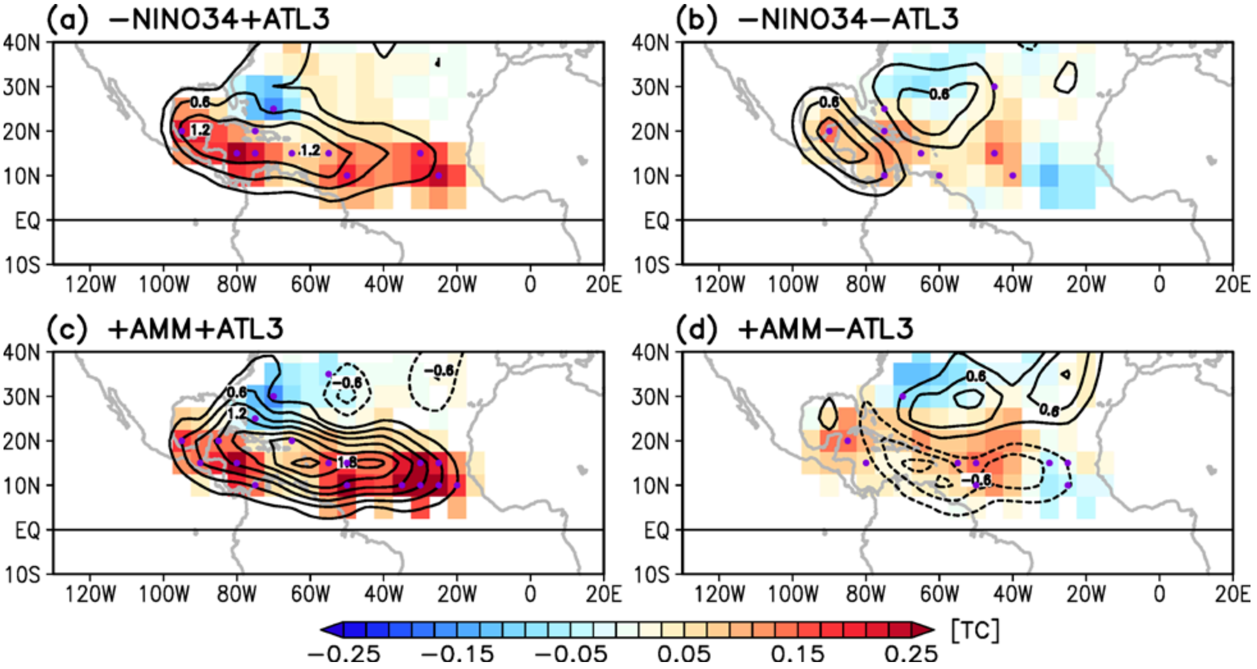

Fig. S5. Partial regression analysis showing the combined impacts of Atlantic Niño/Niña, La Niña, and positive Atlantic meridional mode on Atlantic tropical cyclone activity. (a) Linear summation of the partial regression of tropical cyclone genesis (TC, shaded), and track density (black contours) onto Atlantic Niño (ATL3) and Niño 3.4 (NINO34) indices. (b) is the same as (a) but for linear subtraction. (c) is the same as (a) but for ATL3 and Atlantic meridional mode (AMM) indices. (d) is the same as (b) but for ATL3 and AMM. Purple dots indicate where TC genesis regressions are significant above the 95% confidence level based on a Student's t-test.

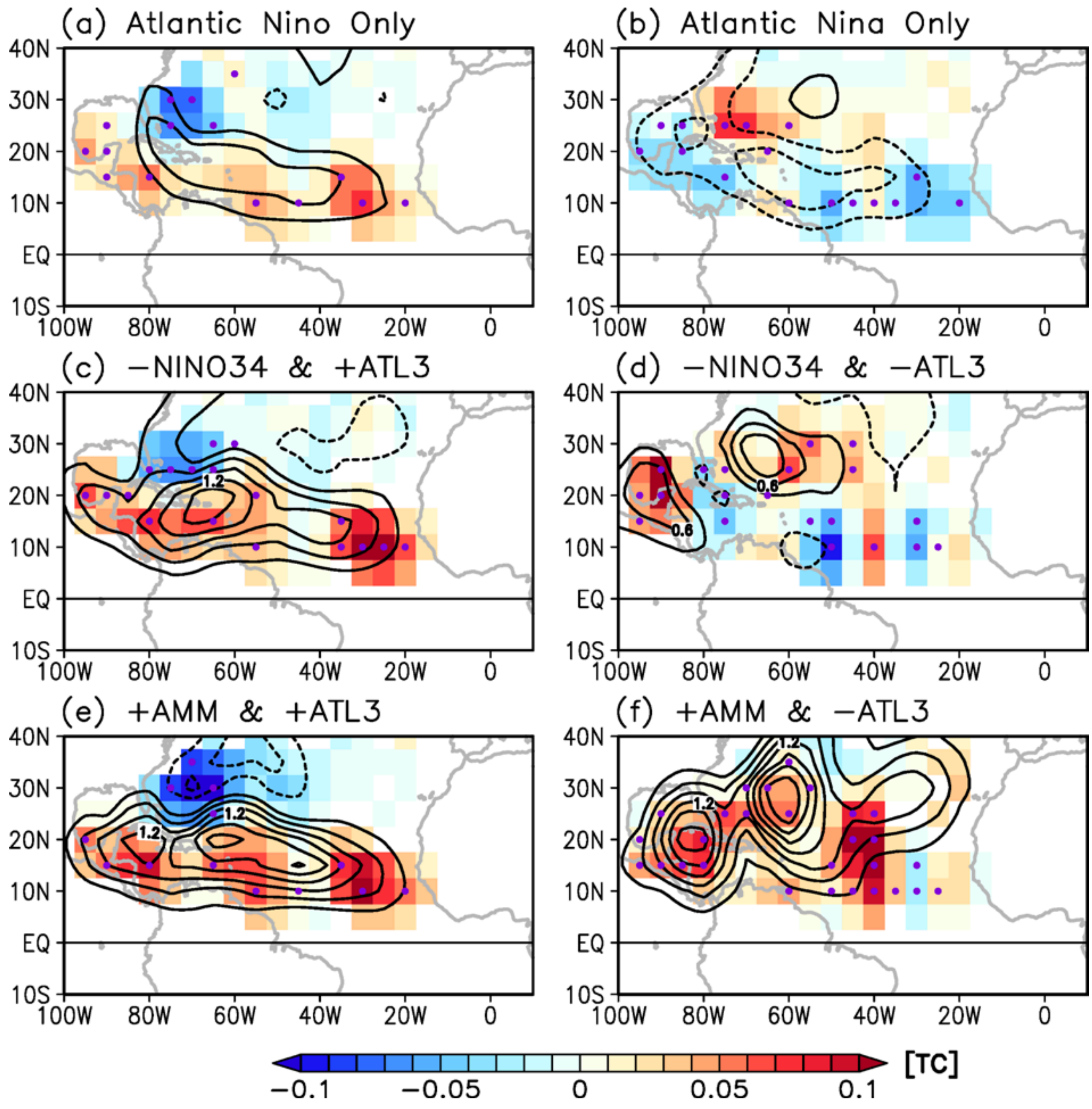

Fig. S6. Composite analysis of the combined impacts of Atlantic Niño/Niña, La Niña, and positive Atlantic meridional mode on Atlantic tropical cyclone activity. Spatial patterns of anomalous tropical cyclone (TC) genesis (shaded, per year) and track density (black contours, intervals of 0.4 TCs per year) composites during (a) Atlantic Niño only and (b) Atlantic Niña only cases. Purple dots indicate where TC genesis anomalies are significant above the 90% confidence level based on a Student's t-test. TC genesis and track density are spatially smoothed

to aid visual comparison. (c) and (d) are spatial patterns of anomalous TC genesis (shaded, per year) composites during La Niña & Atlantic Niño and La Niña & Atlantic Niña, respectively. (e) and (f) are the same as (c) and (d) but for TC genesis during positive Atlantic meridional mode (AMM) & Atlantic Niño, and positive AMM & Atlantic Niña. Note that Atlantic Niño/Niña, El Niño/La Nina, and (+)/(-) AMM are identified when their corresponding indices exceed 0.5×standard deviation during June-November or are less than -0.5×standard deviation.
